# Biology and pathophysiology of the amyloid precursor protein

**DOI:** 10.1186/1750-1326-6-27

**Published:** 2011-04-28

**Authors:** Hui Zheng, Edward H Koo

**Affiliations:** 1 Huffington Center on Aging and Department of Molecular & Human Genetics, Baylor College of Medicine, Houston, TX 77030, USA; 2 Department of Neurosciences, University of California, San Diego, La Jolla, CA 92093, USA

## Abstract

The amyloid precursor protein (APP) plays a central role in the pathophysiology of Alzheimer's disease in large part due to the sequential proteolytic cleavages that result in the generation of β-amyloid peptides (Aβ). Not surprisingly, the biological properties of APP have also been the subject of great interest and intense investigations. Since our 2006 review, the body of literature on APP continues to expand, thereby offering further insights into the biochemical, cellular and functional properties of this interesting molecule. Sophisticated mouse models have been created to allow in vivo examination of cell type-specific functions of APP together with the many functional domains. This review provides an overview and update on our current understanding of the pathobiology of APP.

## Introduction

Alzheimer's disease (AD) is the most common cause of dementia and neurodegenerative disorder in the elderly. It is characterized by two pathological hallmarks: senile plaques and neurofibrillary tangles, as well as loss of neurons and synapses in selected areas of the brain. Senile plaques are extracellular deposits composed primarily of amyloid β-protein (Aβ), which is a 40-42 amino acid long peptide derived by proteolytic cleavages of the amyloid precursor protein (APP), with surrounding neuritic alterations and reactive glial cells. Aβ has taken a central role in Alzheimer's disease research for the past two decades in large part because of the amyloid cascade hypothesis which posits that Aβ is the common initiating factor in AD pathogenesis. Because of this, the processing of APP and generation of Aβ from APP have been areas of substantial research focus by a large number of laboratories. By comparison, whether full-length APP or other non-Aβ APP processing products play a significant role in AD or contribute to other neurological disorders has received somewhat less consideration. For example, it is unclear if the mutations in the APP gene found in the hereditary form of familial AD and the related hereditary amyloid angiopathy with cerebral hemorrhage (http://www.molgen.ua.ac.be/ADMutations/) are pathogenic solely because of perturbed Aβ properties. However, increasing evidence supports a role of APP in various aspects of nervous system function and, in view of the recent negative outcome of clinical trials targeting Aβ production or clearance, there is renewed interest in investigating the physiological roles of APP in the central nervous system (CNS) and whether perturbation of these activities can contribute to AD pathogenesis.

This review will update some of the recent findings on the physiological properties of APP. We start with a general overview of APP. Because APP consists of multiple structural and function domains, we will focus our review by addressing the properties of the full-length APP as well as APP extracellular and intracellular domains. Finally, we provide an update on the current knowledge concerning the APP function in vivo, especially recent findings from the *APP *conditional knockout mice and knock-in alleles expressing various APP domains. For discussions on the pathophysiology of Aβ, there are many excellent reviews that summarize this area in detail but is otherwise beyond the scope of this article.

### A. APP Overview

#### a) The APP Family

**Figure 1:**
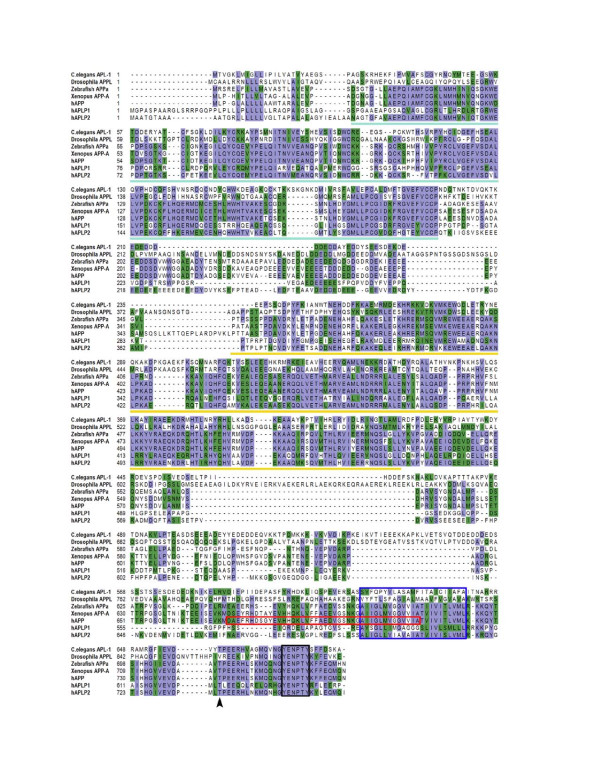
**Comparison of protein sequences of *C. elegans *APL-1, *Drosophila *APPL, Zebrafish APPa, Xenopus APP-A and the human APP, APLP1 and APLP2**. Purple sequences indicate identical homology while green references similar amino acids. Homologous regions include the E1 domain (light blue line), E2 domain (yellow line) and sequences within the C-terminus such as the conserved Thr site (arrow head) and YENPTY motif (black box). The transmembrane domain and Aβ sequence are noted by the blue and red boxes respectively.

APP is a member of a family of conserved type I membrane proteins. The APP orthologs have been identified in, among others, *C. elegans *[1], *Drosophila *[2,3], Zebrafish [4] and *Xenopus Laevis *[5,6]. Three APP homologs, namely *APP *[7,8], APP like protein 1 (*APLP1*) [9] and 2 (*APLP2*) [10,11], have been identified in mammals (Figure 1). These proteins share a conserved structure with a large extracellular domain and a short cytoplasmic domain. There are several conserved motifs, including the E1 and E2 domains in the extracellular region and the intracellular domain, the latter exhibiting the highest sequence identity between APP, APLP1 and APLP2. Of interest, the Aβ sequence is not conserved and is unique to APP. Additionally, the *APP *and *APLP2 *genes, but not *APLP1*, were identified in *Xenopus Laevis*, suggesting that the first gene duplication resulted in *APP *and preAPLP in the evolution of the *APP *superfamily, prior to the separation of mammals and amphibians [12]. Thus, *APLP1 *diverged from the *APLP2 *gene such that *APLP1 *does not contain two additional exons present in both *APP *and *APLP2*, one of which encodes a Kunitz-type protease inhibitor domain. With this history, it is not surprising that *APLP1 *is found only in mammals and, unlike *APP *and *APLP2*, it is expressed only in brain. However, given the sequence identity between the three genes, it is also not unexpected that the mammalian APP homologs play redundant activities in vivo (discussed in "The in vivo Function of APP"). The functional conservation of APP across species is also documented by the partial rescue of the *Drosophila Appl *null behavioral phenotype by human APP [3]. These observations indicate that the conserved motifs, rather than the non-conserved Aβ sequence, likely underline the physiological functions among the APP species.

#### b) APP Expression

The mammalian APP family of proteins is abundantly expressed in the brain. Similar to *Drosophila Appl *[13], *APLP1 *expression is restricted to neurons. However, although highly enriched in the brain, *APP *and *APLP2 *are ubiquitously expressed outside of the brain. The human *APP *gene, located on the long arm of chromosome 21, contains at least 18 exons [14,15]. Alternative splicing generates APP mRNAs encoding several isoforms that range from 365 to 770 amino acid residues. The major Aβ peptide encoding proteins are 695, 751, and 770 amino acids (referred to as APP695, APP751 and APP770). APP751 and APP770 contain a domain homologous to the Kunitz-type serine protease inhibitors (KPI) in the extracellular sequences, and these isoforms are expressed in most tissues examined. The APP695 isoform lacks the KPI domain and is predominately or even exclusively expressed in neurons and accounts for the primary source of APP in brain [16]. For example, there is a burst of increased expression of APP695 during neuronal differentiation. However, following brain injury, expression of the APP751/770 isoforms is substantially increased in astrocytes and microglial cells [17,18]. The reason and functional significance for this apparent tissue-specific alternative splicing is poorly understood.

#### c) APP Processing

**Figure 2:**
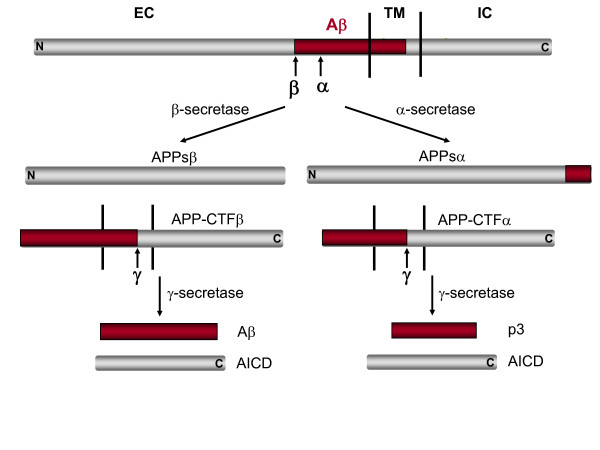
**Schematic diagram of APP processing pathways (not drawn to scale)**. Aβ domain is highlighted in red. For simplicity, only one cleavage site is shown for each enzyme. EC: extracellular; TM: transmembrane; IC: intracellular.

APP is processed in the constitutive secretory pathway and is post-translationally modified by N- and O-glycosylation, phosphorylation and tyrosine sulfation (reviewed in [19]). Full-length APP is sequentially processed by at least three proteases termed α-, β- and γ-secretases (Figure 2). Cleavage by α-secretase or β-secretase within the luminal/extracellular domain results in the shedding of nearly the entire ectodomain to yield large soluble APP derivatives (called APPsα and APPsβ, respectively) and generation of membrane-tethered α- or β-carboxyl-terminal fragments (APP-CTFα and APP-CTFβ). The APP-CTFs are subsequently cleaved by γ-secretase to generate either a 3 kDa product (p3, from APP-CTFα) or Aβ (from APP-CTFβ), and the APP intracellular domain (AICD).

The major neuronal β-secretase is a transmembrane aspartyl protease, termed BACE1 (β-site APP cleaving enzyme; also called Asp-2 and memapsin-2) [20-24], and cleavage by BACE1 generates the N-terminus of Aβ. There is an alternative BACE (β') cleavage site following Glu at position +11 of the Aβ peptide [25]. In addition, there is a BACE2 homolog which is expressed widely but does not appear to play a role in Aβ generation as it appears to cleave near the α-secretase site [26,27]. Of note, cathepsin B has also been proposed to act as a β-secretase [28,29], but whether generation of Aβ in brain requires the coordinated action of both BACE1 and cathepsin B is not known but unlikely given the near total loss of Aβ in BACE1 deficient mice [23,24,30].

While cleavage at the β-site is specific to BACE1 and possibly cathepsin B, it was initially believed that a number of proteases, specifically members of the ADAM (a disintegrin and metalloprotease) family of proteases including ADAM9, ADAM10 and ADAM17 are candidates for the α-secretase (reviewed in [31]). It was reported that APP α-secretase cleavage can be stimulated by a number of molecules, such as phorbol ester or via protein kinase C activation, in which case, this so-called regulated cleavage is mediated by ADAM 17, also called TACE (tumor necrosis factor α-converting enzyme) [32,33]. However, recent studies indicated that constitutive α-secretase activity is likely to be mediated by ADAM10 [34]. Interestingly, ADAM10 is transcriptionally regulated by sirtuins [35], thus providing a mechanism where augmentation of α-secretase activity competes for β-secretase cleavage to lower generation of full length Aβ peptide. However, it should be noted that cleavage of APP by α-secretase processing only precludes the formation of an intact full length Aβ peptide. Although this latter event is commonly called the non-amyloidogenic pathway, it is unfortunately a bit of a misnomer because truncated Aβ (p3 peptide) from 17-42 is also deposited in brains of AD and Down Syndrome patients [36-38], indicating that shorter Aβ peptides starting at the α-secretase site may contribute to some aspects of AD-associated amyloid pathology [39,40].

As regards to γ-secretase cleavage that releases Aβ from the membrane, this activity is executed by a high molecular weight complex consisting of presenilin (PS), nicastrin, anterior pharynx defective (APH1) and presenilin enhancer (PEN2) (reviewed in [41,42]). Although these four proteins form the mature γ-secretase complex, it appears that the core γ-secretase activity resides within presenilin itself functioning as an aspartyl protease [43,44]. In addition to generating Aβ peptides of different lengths, γ-secretase appears to cleave APP in multiple sequential steps [45-47]. An initial cleavage, termed ε-cleavage, taking place 3-4 residues from the cytoplasmic membrane face begins this process [48,49]. Elegant studies by Ihara and colleagues [50-53] have led to a model whereby sequential cleavages taking place every three residues along the α-helical face of the transmembrane domain of APP shortens the C-terminus to ultimately result in the release of Aβ.

It is worth mentioning that none of the secretases have unique substrate specificity towards APP. Besides APP, several transmembrane proteins such as growth factors, cytokines and cell surface receptors and their ligands, undergo ectodomain shedding by enzymes with α-secretase activity (see [54] for an overview). The relatively low affinity of BACE1 toward APP led to the suggestion that APP is not its sole physiological substrate. Indeed, neuregulin-1 (NRG1) now appears to be a bona fide substrate of BACE1 such that the shedding of NRG1 initiated by BACE1 cleavage would direct Schwann cells to myelinate peripheral nerves during development [55,56]. Similarly, γ-secretase has been reported to cleave more than 50 type I membrane proteins in addition to APP (reviewed by [57]), an event that requires an initial ectodomain shedding event, usually by α-secretase-mediated cleavage. While this cleavage in some cases has been demonstrated to initiate intracellular cell signaling, as exemplified by the γ-secretase dependent Notch activation, whether this also applies to APP and other γ-secretase substrates remains unconfirmed (see below and discussed in [58]).

### B. The Full-length APP

#### a) Cell Surface Receptor

Ever since the cloning of APP cDNA, APP has been proposed to function as a cell surface receptor. Further, the analogy between the secondary structures and proteolytic processing profiles between the Notch receptor and APP also suggests that APP could function as a cell surface receptor similar to Notch (reviewed in [59]). In support of this hypothesis, Yankner and colleagues reported that Aβ could bind to APP and thus could be a candidate ligand for APP [60], a finding that has been replicated by others [61]. Another piece of evidence came from Ho and Sudhof (2004) who showed that the APP extracellular domain binds to F-spondin, a neuronally secreted glycoprotein, and this interaction regulates Aβ production and downstream signaling [62]. Similarly, the Nogo-66 receptor has been shown to interact with the APP ectodomain and by which means affect Aβ production [63]. Another interacting protein recently reported is Netrin-1, a soluble molecule with multiple properties including axonal guidance through chemoattraction and tumorigenesis [64]. In this instance, addition of netrin-1 to neuronal cultures led to reduction in Aβ levels but also increased APP-Fe65 complex formation, thus suggesting a role in cell signaling (see below). Recently, work from the D'Adamio group showed that BRI2 could function as a putative ligand or co-receptor for APP and modulates APP processing [65,66]. Finally, the fact that the extracellular domains of the APP family of protein could potentially interact in *trans *(discussed below) suggest that APP molecules can interact in a homophilic or heterophilic manner between two cells. Overall, although a number of APP interacting proteins have been identified, it is unclear whether any of the candidates are *bona fide *ligands and definitive evidence supporting a physiological role of APP to function as a cell surface receptor is still lacking.

#### b) Cell and Synaptic Adhesion

The E1 and E2 regions in the extracellular domain of APP have been shown to interact with extracellular matrix proteins and heparin sulfate proteoglycans (reviewed in [67]), supporting its role in cell-substratum adhesion. The same sequences have also been implicated in cell-cell interactions. Specifically, X-ray analysis revealed that the E2 domain of APP could form parallel or antiparallel dimers [68], the latter structure would imply that there is a potential to function in trans-cellular adhesion. Indeed, cell culture studies support the homo- or hetero-dimer formation of the APP family members, and trans-dimerization was shown to promote cell-cell adhesion [69]. It was further shown that heparin binding to the E1 or E2 region would induce the formation of APP dimerization [70]. Besides the E1 and E2 regions, recent studies suggest that homodimerization can be promoted by the GxxxG motif near the luminal face of the membrane [71,72]. Interestingly, mutagenesis of the glycine residues in this motif resulted in production of truncated Aβ peptides of 34, 35, and 38 amino acids in length [71]. On the other hand, it is unclear whether these changes in Aβ generation are strictly related to APP dimerization because forced dimerization of APP with a bifunctional cross-linking agent did not lead to the same changes in Aβ profile [73]. In addition, while trans-dimerization would be expected to play a role in cell-cell interactions or adhesion, it is less clear what the cellular consequences of cis-homodimerization of APP are, aside from the alterations in Aβ peptides noted earlier. One possible role of dimerization is through downstream activity of the AICD peptide that is released after ε-cleavage, but support for this idea remains controversial. Interestingly though, using various reporter constructs, the subcellular localization of dimerized APP and APLP2 was reported to be different to that of APLP1 [74], suggesting that there are subtle functional roles in homo- or heterodimerization of the APP gene family that remain to be elucidated. Lastly, near the beginning of the Aβ sequence (and near the C-terminus of APPs) is a "RHDS" tetra-peptide motif that also appears to promote cell adhesion. It is believed that this region acts in an integrin-like manner by its homology to the "RGD" sequence [75]. In this regard, it is interesting that APP colocalizes with integrins on the surface of axons and at sites of adhesion [76,77]. In support of these earlier observations, it was recently shown that APP and integrin-β1 do interact [78] and that siRNA mediated silencing of APP during development led to defects in neuronal migration that may be related to cell adhesion [79], potentially to extracellular matrix proteins, with or without participation by integrins.

More compelling evidence of trans-APP dimerization was recently obtained in a primary neuron/HEK293 mixed culture assay. In this culture system, it was reported that trans-cellular APP/APP interaction induces presynaptic specializations in co-cultured neurons [80]. These studies identified APP proteins as a novel class of synaptic adhesion molecules (SAM) with shared biochemical properties as neurexins (NX)/neuroligins (NL), SynCAMs, and leucine-rich repeat transmembrane neuronal proteins (LRRTM) [81-86]. Like NX/NL and SynCAM-mediated synaptic adhesion in which extracellular sequences engage in trans-synaptic interactions and the intracellular domains recruit pre- or postsynaptic complexes (reviewed in [87]), both the extracellular and intracellular domains of APP are required to mediate the synaptogenic activity. Interestingly, using an affinity tagged APP molecule expressed in transgenic mice, the identified "APP-interactome" consisted of many proteins, such as Bassoon and neurexin, that are synaptic in localization [88]. Whether APP trans-synaptic interaction is involved in the recruitment of these synaptic molecules and whether APP coordinates with other synaptic adhesion complexes such as neurexin are interesting questions that warrant further investigation.

### C. The APP Ectodomain

Various subdomains can be assigned to the APP extracellular sequences based on its primary sequences and structural studies (Figure 1) (reviewed in [89,90]). These include the E1 domain, which consists of the N-terminal growth factor-like domain (GFLD) and the metal (copper and zinc) binding motif, the KPI domain present in APP751 and APP770 isoforms, and the E2 domain which includes the RERMS sequence and the extracellular matrix components. We address below the functional studies associated with the APP extracellular domain.

#### a) Synaptotrophic and Neuroprotective Functions

A number of publications have pointed to a neurotrophic role of the APP extracellular domain in both physiological and pathological settings, and this function may be linked to its adhesive properties described above either in its full-length form or as a secreted molecule (i.e. APPs) following ectodomain shredding. Thus, APP may exert these activities in both autocrine and paracrine fashions. Of note, APP undergoes rapid anterograde transport and is targeted to the synaptic sites [16,91-93], where levels of secreted APP coincide with synaptogenesis [94]. APP expression is upregulated during neuronal maturation and differentiation [95,96]. Its expression is also induced during traumatic brain injury both in the mammalian system and in *Drosophila *[18,97-99].

The crystal structure of the E1 domain shows similarities to known cysteine-rich growth factors and thus this domain in the N-terminus of APP has been linked to growth factor-like domain (GFLD) that is seen in the epidermal growth factor receptor [100]. One of the earliest indications of APP function came from the observation that assessing fibroblasts treated with an antisense *APP *construct grew slower and the growth retardation can be restored by treatment with secreted APPs [101]. The active domain was subsequently mapped to a pentapeptide domain "RERMS" in the E2 domain [102]. The activity is not limited to fibroblasts as infusion of this pentapeptide or APPsα into the brain resulted in increased synaptic density and better memory retention, while injection of APP antibodies directly into the brain led to impairment in behavioral tasks in adult rat [103]. Application of APPsα resulted in reduced neuronal apoptosis and improved functional recovery following traumatic brain injury (TBI) [103-105]; it also antagonized dendritic degeneration and neuronal death triggered by proteasomal stress [106]. These findings are corroborated by additional reports showing that reduction or loss of APP is associated with impaired neurite outgrowth and neuronal viability in vitro and synaptic activity in vivo [107-109]. Recent studies have further substantiated these early findings, showing for example that APPs regulates NMDA receptor function, synaptic plasticity and spatial memory [110], and that the growth promoting property may be mediated by the down-regulation of CDK5 and inhibition of tau hyperphosphorylation by APPsα [111]. Finally, a number of studies have reported the effects of APPsα on stem cells. Caille et al. first demonstrated the presence of binding sites for APPs in epidermal growth factor (EGF)-responsive neural stem cells in the subventricular zone in the adult rodent brain [112]. In this context, APPsα acts as a co-factor with EGF to stimulate the proliferation of these cells both in neurospheres in culture and *in vivo*. Subsequently, it was reported that APPs promoted neurite outgrowth in neural stem cells where APLP2 but not APLP1 was redundant to APP [113]. However and intriguingly, stem cells from APP/APLP1/APLP2 triple knockout embryos did not show any defects in neuronal differentiation *in vitro *[114]. Furthermore, in APP transgenic mice, overexpression of wild type APP resulted in decreased neurogenesis but promoted survival of newly generated cells [115]. At the moment, it is unclear how all these findings can be reconciled in a parsimonious picture of APP trophic functions.

Li et al. recently uncovered a novel role for APPs to regulate gene expression likely through binding to an unknown receptor [116]. In particular, they identified transthyretin (TTR) and Klotho as downstream targets of APP that are mediated by APPsβ. These targets are of direct relevance to AD as TTR has been shown to bind and sequester Aβ [117-119], and Klotho has been extensively implicated in the aging process [120-122]. The regulation of TTR and Klotho expression by APPsβ offers the intriguing possibility for a self-protective mechanism in the APP processing pathway to counter the production and toxicity of Aβ during aging. Because APPs levels have been reported to be reduced in individuals with AD [123-126], the results support the view that the loss of trophic activity or the defence mechanism of APPs may contribute at least in part to the neurodegeneration in AD.

Lastly and perhaps related to the growth promoting property of APP, an area that has come to light concerning APP function involves carcinogenesis, coinciding with the recent observation of an inverse association between cancer and AD [127]. Previous studies have reported an up-regulation of APP in various solid tumors. The reason for this is unclear but a recent study demonstrated that APP plays a role in growth of cancer cells [128]. Whether this potential tumorigenic activity involves adhesion, trophic properties of APPs, or cell signaling remain to be established.

#### b) Axonal Pruning and Degeneration

Whereas ample evidence support a role of APPsα in synaptotrophic and neuroprotective activities, APPsβ is known to be much less active or even toxic (reviewed in [129]). The differential activities between APPsα and APPsβ are difficult to comprehend considering that there are only 17 amino acid differences between the two isoforms and sequences implicated in trophic activities are mapped outside this region and common to both isoforms. The most striking finding related to differences between APPsα and APPsβ came from Nikolaev et al. who reported that, under trophic withdrawal conditions, APPsβ but not APPsα undergoes further cleavage to produce an N-terminal ~35 kDa derivative (N-APP), which binds to DR6 death receptor and mediates axon pruning and degeneration [130]. The authors attempted to link this pathway to both axonal pruning during normal neurodevelopment and neurodegeneration occurring in AD. However, by using recombinant APPsβ in vitro and by creating APPsβ knockin mice in vivo [116], Li et al. demonstrate that APPsβ is highly stable and that APPsβ fails to correct the nerve sprouting phenotype of the *APP/APLP2 *null neuromuscular synapses (discussed in detail under "APP knockin mice"). Therefore, the biological and pathogenic relevance of the APPsβ/DR6 pathway outside of the trophic withdrawal paradigm requires further examination.

### D. The APP Intracellular Domain

The high degree of sequence conservation between the intracellular domains of APP proteins predicts that it is a critical domain mediating APP function. Indeed, this relatively short cytoplasmic domain of 47 amino acid residues contains one well described phosphorylation site as well as multiple functional motifs and multiple binding partners that contribute to trafficking, metabolism, and possibly cell signaling functions of APP.

#### a) Phosphorylation and Protein-Protein Interaction

APP can be phosphorylated at multiple sites in both extracellular and intracellular domains (reviewed by [131]). Among these, the phosphorylation at the threonine residue within the VT^668^PEER motif (Thr^668^) in the APP intracellular domain (Figure 1) has received most of the attention. Several kinases have been implicated in this phosphorylation event, including cyclin-dependent kinase 5 (CDK5), c-Jun N-terminal kinase 1 (JNK1) and JNK3, CDK1/CDC2 kinase and GSK3β [132-135]. Phosphorylation at this residue has been reported to result in several outcomes. First, it has been implicated to regulate APP localization to the growth cones and neurites [134,136], a finding consistent with the preferential transport of Thr^668 ^phosphorylated APP to nerve terminals [137]. Second, phosphorylation at Thr^668 ^has been reported to contribute to Aβ generation, a finding consistent with an increase of Thr^668 ^phosphorylated APP fragments in brains of AD individuals [138]. Third, Thr^668 ^phosphorylation leads to resistance of APP to be cleaved by caspases between Asp^664 ^and Ala^665 ^residues, an event that has been proposed to result in increased vulnerability to neuronal death (see below). Fourth, phosphorylation at Thr^668 ^leads to a conformational change in the APP cytoplasmic domain such that interaction with the cytoplasmic adaptor Fe65 through the distal YENPTY motif [139] is altered, thereby affecting the proposed nuclear signaling activity of the APP-Fe65 complex [140]. As the YENPTY motif has been shown to bind several other cytosolic adaptor proteins, it is not surprising then that Thr^668 ^phosphorylation has also been reported to modulate APP interaction with Mint-1/X11a [141]. Lastly, following phosphorylation, it has been shown that the peptidyl-propyl cis/trans isomerase Pin1 catalyzes the cis to trans isomerization of the Thr^668^-Pro^669 ^bond and this is predicted to alter APP conformation [142], possibly related to the Fe65 or Mint-1/X11a interaction with APP. In support of this idea, it was shown that loss of Pin1 in mice resulted in accumulation of hyperphosphorylated tau and increased Aβ levels [142,143], two features that should accelerate AD pathology in the brain. Nevertheless, knockin mice replacing the Thr668 with a non-phosphorylatable Ala residue did not result in substantive changes in either APP localization or in the levels of Aβ in brain [144], raising the question whether Thr^668 ^phosphorylation plays a significant role in regulating APP trafficking and Aβ generation *in vivo*.

In addition to Thr^668 ^phosphorylation, the highly conserved APP intracellular domain has been shown to bind to numerous proteins (reviewed in [145,146]). Of particular interest and relevance to this review, the Y^682^ENPTY motif is required to interact with various adaptor proteins, including Mint-1/X11a (and the family members Mint-2 and Mint-3, so named for their ability to interact with Munc18), Fe65 (as well as Fe65 like proteins Fe65L1 and Fe65L2) and c-Jun N-terminal kinase (JNK)-interacting protein (JIP), through the phosphotyrosine-binding (PTB) domain. The Y^682 ^has been shown to modulate APP processing in vivo [147]. Of interest is the finding that Fe65 acts as a functional linker between APP and LRP (another type I membrane protein containing two NPXY endocytosis motifs) in modulating endocytic APP trafficking and amyloidogenic processing [148].

#### b) Apoptosis

In contrast to the trophic activities of the soluble APP ectodomain, there are also a number of papers demonstrating the cytotoxic properties of β-secretase cleaved APP CTF (or C99), especially following overexpression [149-151]. The mechanism by which APP CTF is cytotoxic is unclear but one pathway may be through AICD released from APP CTF following ε-cleavage. Normally, AICD exists in very low levels in vivo but can be stabilized when Fe65 is overexpressed [152-154]. In cultured cells, overexpression of AICD led to cell death [154-156]. In transgenic mice overexpressing an AICD construct, there was activation of GSK-3β but no overt neuronal death [157,158], findings not replicated in a subsequent study however [159]. Interestingly, in mice expressing both AICD and Fe65, neuronal degeneration was observed in old mice together with tau hyperphosphorylation. Furthermore, behavioral abnormalities seen in these animals can be rescued by treatment with lithium, a GSK-3β inhibitor, in line with earlier evidence of activation of GSK-3β [160].

Another aspect of APP CTF mediated cytotoxicity concerns a caspase cleavage site within the cytosolic tail between position Asp^664 ^and Ala^665 ^[161]. In cell culture systems, loss of this caspase site by mutating the Asp^664 ^to Ala (D664A) resulted in an attenuation of APP C99 associated cytotoxicity. It has been proposed that release of the smaller fragments (C31 and Jcasp) from AICD after cleavage at position 664 results in the generation of new cytotoxic APP related peptides [162]. Thus, overexpression of either C31 or Jcasp, both derived from AICD, have resulted in cytotoxicity. Consistent with these *in vitro *findings, in an APP transgenic mouse line in which the caspase site is mutated to render APP noncleavable, the predicted Aβ-related phenotypes in brain (synaptic, behavior, and electrophysiological abnormalities) were absent in spite of abundant amyloid deposits [163,164]. Therefore, these initial observations indicated that the release of the smaller fragments (C31 or Jcasp) after caspase cleavage of C99 may result in cell death in a manner independent of γ-secretase [165]. However, analysis of another line of APP D664A transgenic mice with substantially higher APP expression failed to replicate the earlier findings [166], but the wide differences in expression of the transgene and resultant Aβ levels between the two transgenic mouse lines is such that the comparisons may be invalid [167]. In sum, there are at present several potential mechanisms whereby APP may contribute to neurotoxicity: via γ-secretase cleavage to release AICD or via alternative cleavage of the APP C-terminus to release other cytotoxic peptides. Whether these APP fragments contribute to *in vivo *neuronal death in AD pathogenesis remain to be established.

#### c) Cell Signaling

As mentioned previously, in addition to γ-secretase cleavage that yields Aβ40 and Aβ42, presenilin-dependent proteolysis appears to begin at the ε-site (Aβ49) close to the membrane-intracellular boundary [46,48,49]. Thus the ε-cleavage of APP may represent the primary or initial presenilin-dependent processing event. This is important because this cleavage releases AICD in a manner highly reminiscent of the release of the Notch intracellular domain (NICD) after γ-secretase processing, the latter being an obligatory step in Notch mediated signaling (reviewed in [59]). The predominant ε-cleavage releases AICD of 50 amino acids in length (CTF50-99), beginning with a Val residue. APP mutations that shift Aβ production in favor of Aβ42 would lengthen the AICD by one amino acid (CTF 49-99), now beginning with a Leu residue. This is of some interest because it has been pointed out that the N-end rule guiding protein stability through ubiquitination states that Val is a stabilizing residue while Leu is destabilizing (Reviewed in [168]). NICD, the intracellular domain derived from the Notch receptor, appears to follow this principle experimentally. If this situation applies to AICD, then there could be a different regulatory mechanism at play regarding AICD mediated cell signaling or in cell death. Furthermore, recent studies have suggested that AICD generation is in part dependent on whether APP was previously cleaved by α- or β-secretase, indicating yet another layer of regulation [169,170]. Nonetheless, AICD is indeed very labile and, as mentioned previously, can be stabilized by Fe65 [153], a finding seen both in the in vitro and in vivo settings. A good deal of excitement followed the first report in which by using a heterologous reporter system, AICD was shown to form a transcriptionally active complex together with Fe65 and Tip60 [157,171]. This finding appeared to validate the notion that AICD is transcriptionally active, much like NICD. Scheinfeld et al. proposed a JIP-1 dependent transcriptional activity of AICD [172]. However, subsequent analyses have suggested that the earlier view may be too simplistic and incomplete. First, follow up studies by Cao et al. showed that AICD facilitates the recruitment of Fe65 but its nuclear translocation *per se *is not required [173]. Second, PS-dependent AICD production is not a prerequisite for the APP signaling activity, as it proceeds normally in PS null cells and by PS inhibitor treatment [174]. Instead, the authors provide an alternative pathway for this activity that involves Tip60 phosphorylation. Third, a later report documented that the proposed signaling activity is actually executed by Fe65 and that APP is not required altogether [175]. Lastly, Giliberto et al. reported that mice transgenic for AICD in neuronal cells are more susceptible to apoptosis. However, analysis of the basal transcription showed little changes in mice expressing AICD in the absence of Fe65 overexpression, leaving open the possibility that transcription may be influenced in a regulated fashion [176].

Regardless of the mechanism by which AICD may activate signaling pathways, a trans-activating role of the APP/Fe65/Tip60 complex has been consistently documented, at least in overexpression systems. However, these efforts have led to decidedly mixed results. A number of genes have been proposed to date including KAI [177], GSK3β [158,178], neprilysin [179], EGFR [180], p53 [181], LRP [182], APP itself [183], and genes involved in calcium regulation [184] and cytoskeletal dynamics [185]. However, the validity of these proposed targets have been either questioned or disputed [175,176,186-190]. Thus, at present, a conservative view is that these target genes are indirectly or only weakly influenced by AICD mediated transcriptional regulation.

### E. *In vivo *Function of APP

The *in vivo *gain- and loss-of-function phenotypes associated with the APP family of proteins in model systems (*C. elegans*, *Drosophila *and mice) are consistent with a role of APP in neuronal and synaptic function in both central and peripheral nervous systems. This may be mediated by the APP ectodomain or requires the APP intracellular domain. These findings will be discussed next in the respective animal models.

#### a) C. elegans

The *C. elegans *homolog of APP, APL-1, resembles the neuronal isoform APP695 as there are no known splice variants detected. Similar to APLP1 and APLP2, APL-1 does not contain the Aβ sequence. Nematode development includes four larval stages (L1-L4) after each of which is a molt where a new, larger exoskeleton is formed to accommodate the growth of the larvae. Inactivation of the single *apl-1 *gene leads to developmental arrest and lethality at the L1 stage, likely due to a molting defect [191,192]. In addition, *apl-1 *knockdown leads to hypersensitivity to the acetylcholinesterase inhibitor aldicarb, signifying a defect in neurotransmission [192]. The aldicarb hypersensitivity phenotype and the molting defect were found to be independent of one another, suggesting *apl-1 *contributes to multiple functions within the worm. Surprisingly, both phenotypes were rescued by either a membrane-anchored C-terminal truncation of APL-1 or by the soluble N-terminal fragments, showing that the highly conserved C-terminus is not required to support the viability of the worm [191,192]. This differs from the mammalian system in which the APP C-terminus is essential for viability on a non-redundant background (see discussion under "APP knock-in mice") [116,193]. Although the reason for the distinct domain requirement for *C. elegans *and mouse viability is not clear, it is worth reiterating that the lethality of the *apl-1 *null worm is likely caused by a molting defect not relevant to mammals. Consistent with this interpretation, it is interesting that expression of mammalian APP or its homologs are not able to rescue the *apl-1 *null lethality [191,192], indicating that this worm-specific molting activity is lost during mammalian evolution and that extrapolation of APP function from apl-1 may not be very informative.

#### b) Drosophila

The *Drosophila *APP homolog, APPL, like the worm homolog, does not contain the Aβ sequence and does not undergo alternative splicing. However, in contrast to the *apl-1 *null worm, *Appl*-deficient flies are viable with only subtle behavioral defects such as fast phototaxis impairment [3]. While human APP is not able to rescue the *C. elegans apl-1 *lethality, the behavioral phenotype present in the *Appl *null fly can be partially rescued by transgenic expression of either fly APPL or human APP [3]. Subsequent loss and gain-of-function studies revealed that APPL plays an important role in axonal transport, since either *Appl *deletion or overexpression caused axonal trafficking defects similar to kinesin and dynein mutants [194,195]. Although a similar role for APP in axonal transport of selected cargos has been reported [196-198], the findings have since been challenged by several laboratories [199].

APPL is required for the development of neuromuscular junctions (NMJs), since *Appl *deletion leads to decreased bouton number of NMJs, whereas *Appl *overexpression dramatically increases the satellite bouton number [200]. This activity can be explained by the formation of a potential complex including APPL, the APPL-binding protein dX11/Mint, and the cell adhesion molecule FasII, which together regulate synapse formation [201]. Overexpression of human APP homologs in *Drosophila *revealed a spectrum of other phenotypes, ranging from 1) a blistered wing phenotype that may involve cell adhesion [202], 2) a Notch gain-of-function phenotype in mechano-sensory organs, which reveals a possible genetic interaction of APP and Notch through Numb [203], and 3) a neurite outgrowth phenotype that is linked to the Abelson tyrosine kinase and JNK stress kinase [99]. Although the pathways implicated in each of the phenotypes are distinct, they all seem to require the APP intracellular domain via protein-protein interactions mediated through the conserved YENPTY sequence. These ectopic overexpression studies should be interpreted with caution because APP interacts with numerous adaptor proteins and many of the APP binding partners also interact with other proteins. Therefore, the phenotypes observed by overexpressing APP or APPL could be caused by the disturbance of a global protein-protein interaction network.

Interestingly, similar to the mammalian system, APPL is found to be upregulated in traumatic brain injury and *Appl*-deficient flies suffer a higher mortality rate compared to controls [99], supporting an important activity of APP family of proteins in nerve injury response and repair.

#### c) Mice

##### i. *APP *single knockout mice

Three mouse *APP *alleles, one carrying a hypomorphic mutation and two with complete deficiencies of *APP *have been generated [204-206]. The *APP *null mice are viable and fertile but exhibit reduced body weight and brain weight. Loss of APP results in a wide spectrum of central and peripheral neuronal phenotypes including reduced locomotor activity [204,205,207], reactive gliosis [205], strain-dependent agenesis of the corpus callosum [205,208], and hypersensitivity to kainate induced seizures [209]. Although these phenotypes indicate a functional role of APP in the CNS, the molecular mechanisms mediating these effects remain to be established. Unbiased stereology analysis failed to reveal any loss of neurons or synapses in the hippocampus of aged *APP *null mice [210]. Attempts to examine spine density in *APP *KO mice have yielded mixed results. Using hippocampal autaptic cultures, Priller et al. reported an enhanced excitatory synaptic response in the absence of *APP*, and the authors attributed this effect to the lack of Aβ production [211]. Follow up studies by the same group reported that *APP *deletion led to a two-fold higher dendritic spine density in layers III and V of the somatosensory cortex of 4-6 month-old mice [212]. However, Lee *et al. *found a significant reduction in spine density in cortical layer II/III and hippocampal CA1 pyramidal neurons of one-year old *APP *KO mice compared with WT controls [213]. It is not clear whether differences in age or brain region may contribute to the discrepancy.

The *APP *null mice show impaired performances in Morris water maze and passive avoidance tasks, and the behavioral deficits are associated with a defect in long term potentiation (LTP) [207,210,214,215], the latter may be attributed to an abnormal GABAergic paired pulse depression [215]. Follow up work demonstrated that APP modulates GABAergic synaptic strength by regulating Cav1.2 L-type calcium channel (LTCC) expression and function in stratial and hippocampal GABAergic neurons [216]. APP deficiency leads to an increase in the levels of α1C, the pore forming subunit of Cav1.2 LTCCs and an enhanced Ca2+ current, which in turn results in reduced GABAergic mediated paired-pulse inhibition and increased GABAergic post-tetanic potentiation [216]. A role of APP in calcium regulation is further documented by APP overexpression and knockdown studies in hippocampal neurons which support an Aβ independent role of APP in the regulation of calcium oscillations [217].

Outside of the CNS, *APP *deficient mice display reduced grip strength [205,207]. This is likely due to impaired Ca^2+ ^handling at the neuromuscular junction (NMJ) as functional recordings revealed that *APP *null mice show abnormal paired pulse response and enhanced asynchronous release at NMJ resulting from aberrant activation of voltage gated N- and L-type calcium channels at motor neuron terminals [218]. Taken together, the studies thus provide strong support for the notion that APP plays an important role in Ca^2+ ^homeostasis and calcium-mediated synaptic responses in a variety of neurons, including GABAergic and cholinergic neurons and possibly others, through which it may regulate the neuronal network and cognitive function.

##### ii. *APP*, *APLP1*, *APLP2 *compound knockout mice

The relatively subtle phenotypes of *APP *deficient mice are likely due to genetic redundancies as evidenced by gene knockout studies. While mice with individual deletion of *APP*, *APLP1 *and *APLP2 *are viable, *APP/APLP2 *and *APLP1/APLP2 *double knockout mice or mice deficient in all three APP family members are lethal in the early postnatal period [219,220]. Intriguingly and due to reasons not well understood, the *APP/APLP1 *double null mice are viable [220]. Although the NMJ of *APP *or *APLP2 *single null mice do not show overt structural abnormalities, the *APP*/*APLP2 *double knockout animals exhibit poorly formed neuromuscular synapses with reduced apposition of presynaptic proteins with postsynaptic acetylcholine receptors and excessive nerve terminal sprouting [221]. The number of synaptic vesicles at the presynaptic terminals is reduced, a finding consistent with defective neurotransmitter release. Examination of the parasympathetic submandibular ganglia of the double deficient animals also showed a reduction in active zone size, synaptic vesicle density, and number of docked vesicles per active zone [222].

Interestingly, tissue-specific deletion of *APP *either in neurons or in muscle on *APLP2 *knockout background resulted in neuromuscular defects similar to those seen in global *APP/APLP2 *double null mice, demonstrating that APP is required in both motoneurons and muscle cells for proper formation and function of neuromuscular synapses [80]. The authors propose that this is mediated by a trans-synaptic interaction of APP, a model that gained support by hippocampal and HEK293 mixed culture assays described above [80]. Interestingly, muscle APP expression is required for proper presynaptic localization of CHT and synaptic transmission, suggesting that trans-synaptic APP interaction is necessary in recruiting presynaptic APP/CHT complex [80,223].

Analysis of *APP/APLP1/APLP2 *triple knockout mice revealed that the majority of the animals showed cortical dysplasia suggestive of neuronal migration abnormalities and partial loss of cortical Cajal Retzius cells [224]. Interestingly, this defect is phenocopied in mice doubly deficient in APP binding proteins Fe65 and Fe65L1 [225]. It should be pointed out however, that morphological similarity does not necessarily implicate functional interaction. Indeed, cortical dysplasia with viable penetrance also exists in mice deficient in various other proteins including PS1, β1 and α6 integrins, focal adhesion kinase, α-dystroglycan and laminin α2 (reviewed in [226]).

In sum, the loss-of-function studies present a convincing picture that members of the *APP *gene family play essential roles in the development of the peripheral and central nervous systems relating to synapse structure and function, as well as in neuronal migration or adhesion. These may be mediated either by the full-length protein or by various proteolytic processing products, and may be due to mechanical properties or through activating signaling pathways, or both. The creation of knockin alleles expressing defined proteolytic fragments of APP offers a powerful system to delineate the APP functional domains *in vivo*. These are discussed in the following section.

##### iii. *APP *Knock-in mice

To date, four *APP *domain knock-in alleles have been reported and these express α-secretase (APPsα [227]) or β-secretase (APPsβ [116]) processed soluble APP, the membrane anchored protein with deletions of either the last 15 aa (APPΔCT15 [227]) or 39 aa (APP/hAβ/mutC [193]) of the highly conserved C-terminal sequences of APP, the latter also replaced mouse Aβ with the human sequence and introduced three FAD mutations (Swedish, Arctic, and London) to facilitate Aβ production. The APPsα and APPΔCT15 knock-in mice appeared to rescue a variety of phenotypes observed in *APP *KO mice [227]. For instance, the reduced body and brain weight of *APP *null animals was largely rescued. Behaviorally, the knock-in mice do not exhibit any defects in grip strength or the Morris water maze test. Field recordings of hippocampal slices showed that the LTP deficits observed in 9-12 month-old *APP *KO mice was also absent in both knock-in lines. These findings are in agreement with the large body of literature documenting the synaptotrophic activity of APPsα (refer to "Synaptotrophic and Neuroprotective Functions" above) and that perhaps the predominant function of APP is mediated by APPsα.

Similar to APPsα and APPΔCT15 knock-in lines, the APPsβ and APP/hAβ/mutC mice did not show any overt growth or anatomatical deficits. However and in stark contrast to the aforementioned two knockin mouse lines, crossing these two alleles (APPsβ or anchored APP/hAβ/mutC) to *APLP2*^*-/- *^background failed to rescue the early postnatal lethality and neuromuscular synapse defects of the *APP/APLP2 *KO mice [116,193], suggesting a critical and indispensable role of the conserved C-terminal region of APP in early postnatal development. An essential role of the APP C-terminal domain, specifically the YENPTY motif, in development was demonstrated by the creation of APP knock-in mice in which the Tyr^682 ^residue of the Y^682^ENPTY sequence was changed to Gly (APP^YG^). Crossing the homozygous knock-in mice to *APLP2 *null background showed that the *APP*^YG/YG^/*APLP2*^-/- ^mice exhibit neuromuscular synapse deficits and early lethality similar to *APP/APLP2 *double KO mice [228]. The differences in outcomes in these experiments are difficult to explain but may be related to a more severe phenotype in the *APLP2 *deficient background. Nevertheless, the inability to rescue the NMJ defects by the APP mutants lacking the intracellular domain or expressing the Tyr^682 ^to Gly mutation is compatible with the concept that APP functions as a synaptic adhesion protein. Furthermore, the fact that amyloid deposition can develop in the absence of the APP C-terminal sequences indicates that APP developmental function and amyloidogenesis are differentially regulated and require distinct APP domains [193].

## Concluding Remarks

We hope this review has provided a timely update on what is known and what lies ahead in the field of APP biology. Since the first identification of the APP gene in 1987, the scientific community has worked together to obtain significant insights into the biochemical, cellular and functional properties of APP. It is clear that APP undergoes tightly regulated trafficking and processing and, through either the full-length protein and/or its cleavage products, it mediates synaptogenic and synapotrophic activities in development and during aging. As such, it is reasonable to speculate that misregulation of APP could contribute to the neuronal and synaptic impairment occurring in AD. Many key questions remain to be addressed. These include determining whether APP is a receptor or a ligand and, accordingly, the identities of its respective ligand or receptor. Does APP directly mediate cell signaling or only play a secondary role in gene expression? How is APP function coordinated between its full-length form and the various processing products, and how is it facilitated through its binding partners? Elucidating these questions will undoubtedly reveal novel insights into disease pathogenesis.

## Competing interests

The authors declare that they have no competing interests.

## Authors' contributions

HZ and EHK wrote the manuscript. HZ made the figures. Both authors have read and approved the final manuscript.
